# Refining within-subject biological variation estimation using routine laboratory data: practical applications of the refineR algorithm

**DOI:** 10.1515/cclm-2024-1386

**Published:** 2024-12-30

**Authors:** Eirik Åsen Røys, Kristin Viste, Christopher-John Farrell, Ralf Kellmann, Nora Alicia Guldhaug, Elvar Theodorsson, Graham Ross Dallas Jones, Kristin Moberg Aakre

**Affiliations:** Hormone Laboratory, Department of Medical Biochemistry and Pharmacology, Laboratory Medicine and Pathology, 60498 Haukeland University Hospital, Bergen, Norway; Department of Clinical Science, University of Bergen, Bergen, Norway; Department of Clinical Chemistry, NSW Health Pathology, Liverpool Hospital, Liverpool, NSW, Australia; Department of Clinical Chemistry, and Department of Biomedical and Clinical Sciences, Linköping University, Linköping, Sweden; Department of Chemical Pathology, SydPath, St. Vincent’s Hospital, Darlinghurst, NSW, Australia; Faculty of Medicine, University of New South Wales, Kensington, NSW, Australia; Department of Heart Disease, 60498 Haukeland University Hospital, Bergen, Norway

**Keywords:** indirect methods, laboratory information systems, biological variation

To the Editor,

Within-subject biological variation (CV_I_) refers to the natural fluctuations in an individual’s biological markers over time not accounted for by known physiological effects. Accurate estimation of CV_I_ is pivotal for setting analytical performance specifications and identifying clinically significant changes in repeated laboratory tests. Recent research suggests that using data from laboratory information systems (LIS) offers a practical way to estimate the CV_I_ [1], 2].

One indirect method for estimating CV_I_ involves characterizing a central Gaussian peak from the frequency distribution of ‘result ratios’, the ratios of consecutive results from serial patient measurements [1], 2]. This total variation (CV_Total_) includes the random biological (CV_I_) and relevant analytical variation (CV_A_) of the two measurements. A challenge when estimating CV_I_ from LIS data is to distinguish this peak from pathological or other non-random changes [3]. A recent study showed that the refineR algorithm could determine the result ratio peak and further estimate reference change values (RCV) [4]. RefineR identifies a central ‘Box-Cox normal distribution’ within the data [5]. The Box-Cox transformation is robust and can make a range of either left and right skewed distributions resemble Gaussian distributions [5], which is valuable because ratios for some analytes have skewed distributions. In the current study, we applied the refineR algorithm to Gaussian and skewed distributions to derive indirect-CV_I_ estimates and compare these to CV_I_ values derived from a state-of-the-art biological variation (BV) study [1].

Several aspects must be considered when estimating the CV_I_ from ratios [6]. First, consider the case where two independent measurements, X_1_ and X_2_, follow the same positive Gaussian distribution: X ∼ N (μ_x_, σ_x_), with mean μ_x_ and standard deviation σ_x_, forming the ratio Z = X_1_/X_2_. The distribution of Z does not have a simple mathematical description and can be nearly Gaussian or markedly skewed [2], 4], 6], 7]. In the case of nearly Gaussian ratios, i.e. Z ∼ N (μ_z_, σ_z_), μ_z _= 1 and σ_z _= √2 × σ_x_/μ_x_ are good approximations [1], 2]. Rearranging for σ_x_ gives σ_x _= σ_z_ × μ_x_/√2 and as CV_x _= σ_x_/μ_x_ we get:

(1) $${\text{CV}}_{x}\%=\frac{\sigma_{z}}{\sqrt{2}}\times 100\%$$

In the case of a skewed Z, the approximation is no longer valid, as the skew increases the mean and standard deviation of the ratios. To mitigate this, we can apply the Box-Cox transformation to make Z approximately Gaussian. From the standard deviation of the transformed ratios Z′, the CV% of X can be estimated using formula (1).

Secondly, consider the case where the two independent measurements, X_1_ and X_2_, are drawn from the same lognormal distribution ln(X) ∼ N (μ_x_, σ_x_). By the properties of logarithms, the ratio Z = X_1_/X_2_ corresponds to ln(Z) = ln(X_1_) – ln(X_2_), and ln(Z) will be normally distributed with mean, μ_z _= μ_x_ – μ_x_= 0, and sd, σ_z _= √(σ_x_^2^ + σ_x_^2^) = √2σ_x_. Rearranging for σ_x_ gives σ_x _= σ_z_/√2. σ_x_ can be used to calculate the CV% of a lognormal distribution (ln-CV%) [8]:

(2) $$\text{lnCV}\%=\sqrt{\exp\left(\sigma_{x}^{2}\right)-1}\times 100\%$$

We used Monte Carlo-simulated data to validate the assumption that the standard deviation of a Box-Cox transformed ratio distribution could estimate the CV% and ln-CV%. Two Gaussian or two lognormal distributions were simulated using one million observations, applying refineR to estimate the standard deviation of a Box-Cox transformed ratio distribution. The Box-Cox(σ) was divided by √2 to estimate the CV% or ln-CV% as described above. We compared the input CV% and ln-CV% in the simulation with the estimates from refineR, see Table 1. These results showed that refineR could accurately estimate the CV from a ratio of two Gaussian distributions for CVs ≤15 % and with less accuracy up to CVs of ≤20 %, reflecting the error propagation in ratios for larger Gaussian CVs [9]. The ln-CV could be estimated accurately up to at least 50 %.

**Table 1:** Monte Carlo simulation results comparing the input CV% values of Gaussian or lognormal distributions against the estimated CV% from their respective ratio distributions using refineR. Each simulated ratio distribution contained 1 million ratios, with results averaged across 100 iterations.

Ratio distribution	Input CV%	Estimated CV%
Ratio of Gaussian	2.5	2.5
	5.0	5.0
	7.5	7.5
	10.0	10.0
	12.5	12.4
	15.0	14.8
	17.5	17.1
	20.0	19.4
Ratio of lognormal	5.0	5.0
	10.0	10.0
	15.0	15.0
	20.0	20.0
	25.0	25.0
	30.0	30.0
	35.0	35.0
	40.0	40.0
	45.0	45.0
	50.0	50.0

With this proof of concept validated, we applied our approach to result ratios from LIS data. Here, we used the same dataset as our previous study [4], applying refineR to ratios of repeated patient measurements to estimate the total variability of a single measurement (CV_Total_) using either formula (1) or (2). Briefly, this dataset contained adult patient results (18–110 years) with biomarkers selected to represent a wide range of CV_I_ levels (∼2–50 %): albumin, creatinine, phosphate, cortisone, cortisol, testosterone, androstenedione, 17-hydroxyprogesterone and 11-deoxycortisol extracted from local databases at Haukeland University Hospital from July 1, 2015, to July 1, 2023. The indirect-CV_I_ was then estimated by subtracting long-term CV_A_ from quality controls from the CV_Total_ as:

(3) $${\text{Indirect}\;\text{CV}}_{I}=\sqrt{{{\text{CV}}_{\text{Total}}}^{2}-{{\text{CV}}_{A}}^{2}}$$

The results are shown in Table 2. The 95 % confidence interval for the Box-Cox(σ) and the indirect-CV_I_ was derived using refineR’s bootstrapping capabilities (1,000 repetitions). As in our previous paper [4], we compared these results with the estimated CV_I_ from a local state-of-the-art BV study combining CV-ANOVA with outlier exclusion [1]. This study included 10 weekly measurements from 30 healthy subjects for the same analytes and instrumentation as for the LIS data.

**Table 2:** Indirect CV_I_ estimates obtained from applying the refineR algorithm on a result ratio distribution of data from the laboratory information system and from CV_I_ estimates from a biological variation study [1] using nested-ANOVA.

Measurand	Subgroup	Number of ratios	Long-term CV_A_	Box-Cox(σ) [95 % CI]	Indirect CV_I_ [95 % CI]	CV_I_ [95 % CI]
Albumin	All	949,295	1.8	0.052 [0.052, 0.053]	3.2 [3.2, 3.3]	2.3 [2.1, 2.6]
Creatinine	All	400,152	3.0	0.076 [0.074, 0.077]	4.4 [4.3, 4.6]	4.4 [4.0, 4.8]
Phosphate	All	25,901	1.7	0.141 [0.121, 0.148]	9.8 [8.4, 10.3]	9.5 [8.7, 10.4]
Phosphate	Female	15,426	1.7	0.132 [0.124, 0.137]	9.2 [8.6, 9.6]	8.2 [7.6, 9.8]
Phosphate	Male	10,475	1.7	0.143 [0.125, 0.158]	10.0 [8.6, 11.0]	9.4 [8.3, 11.0]
Cortisone	Male	6,882	7.0	0.202 [0.191, 0.209]	12.5 [11.6, 13.0]	12.6 [11.0, 15.0]
Cortisol	Male	4,983	4.4	0.271 [0.247, 0.310]	18.6 [16.9, 21.4]	15.1 [12.5, 18.6]
Testosterone	Male	1,261	4.0	0.244 [0.178, 0.269]	16.9^a^	15.3^a^ [13.3, 17.6]
					[12.0, 18.8]	
Androstenedione	Male	6,023	5.3	0.334 [0.295, 0.357]	23.0^a^	19.2^a^ [17.1, 22.6]
					[20.2, 24.7]	
17-Hydroxyprogesteron	Male	6,839	4.8	0.351 [0.304, 0.378]	24.7^a^ [21.2, 26.7]	23.0^a^ [20.1, 27.0]
11-Deoxycortisol	Male	6,469	5.3	0.584 [0.546, 0.620]	42.6^a^ [39.6, 45.6]	50.0^a^ [44.9, 61.1]

^a^Calculated as ln-CV.

Although the indirect-CV_I_ estimates were generally higher than those derived from the BV study, they were comparable, with overlapping confidence intervals (Table 2). The differences between the two estimates are likely due to larger preanalytical variability in the LIS data, as these were produced under daily routine conditions, while the BV study was performed in accordance with recommendations from BIVAC [10], minimizing preanalytical variability. Cortisol was the analyte with the greatest relative difference between the estimates, and this probably relates to its pronounced diurnal variation and the effect of even small changes in sample collection timing.

One of the main benefits of the refineR algorithm is that it can effectively separate healthy and pathological ratios [4]. A strength of this study is that the indirect-CV_I_ ratio approach was validated by Monte Carlo simulation and by comparing it with a local BV study. However, the approach has limitations that may affect the accuracy of the CV_I_ estimates: As previously shown, some heavily skewed ratio distributions cannot be Box-Cox transformed to Gaussian [4]. The ratios also do not reveal the underlying distribution’s shape, as Gaussian and lognormal distributions can produce similarly distributed ratios. The errors introduced by choosing a non-applicable formula are minimal for small CV_I_s (<20 %) but will increase if the CV_I_ is large.

A final limitation is that using non-standardized LIS data can produce inaccurate CV_I_ estimates. Care must be taken to minimize known effects such as time of day, effect of dietary intake, and physiological events such as pregnancy or the menstrual cycle. The lack of standardized sampling procedures associated with LIS data may bias the CV_I_ estimates compared with a direct sampling approach, where the preanalytical factors are controlled. Based on the indirect-RCV study [4], we recommend ≥5,000 ratios and a pathological fraction of <30 % for robust indirect-CV_I_ estimates. Datasets commonly used for indirect reference intervals such as outpatient data, wellness checkups, repeated testing in blood donors or unrequested results should be considered.

Despite its limitations, the refineR algorithm provides a valuable, cost-effective tool for laboratories to estimate CV_I_ using readily available data, especially for subgroups with limited direct studies, such as children. To make the refineR approach available to laboratories, we have developed an application at (https://gocrunch.shinyapps.io/CVi_app/) that can estimate the indirect-CV_I_, RCV, and reference intervals from LIS datasets.
